# Beneath Sepsis, Lupus-Like Autoimmunity, and COVID-19: Diagnostic Eclipse in Fatal Intravascular Large B-cell Lymphoma

**DOI:** 10.7759/cureus.111936

**Published:** 2026-07-02

**Authors:** Khalid Shahzad, Muhammad Tanseer Sibtain Raza, Umair Falak, Muhammad Wasim Tariq, Adnan Abbas Nizami

**Affiliations:** 1 Internal Medicine, Our Lady's Hospital, Navan, IRL; 2 Internal medicine, King Edward Medical University, Lahore, PAK; 3 Respiratory Medicine, Army Medical College, Rawalpindi, PAK; 4 Internal Medicine, King Edward Medical University, Lahore, PAK

**Keywords:** autoimmune mimicry, culture-negative sepsis, diagnostic delay, differential for fever of unknown origin, intravascular large b-cell lymphoma (ivlbcl)

## Abstract

Intravascular large B-cell lymphoma (IVLBCL) is a rare extranodal neoplasm in which malignant B cells proliferate predominantly within small blood vessels. Because lymphadenopathy, mass lesions, circulating malignant cells, and specific radiological findings may be absent, IVLBCL can closely mimic other medical conditions, resulting in delayed diagnosis. This case is notable for a prolonged culture-negative sepsis-like presentation with persistent unexplained hypoxemia due to predominant pulmonary microvascular involvement, further obscured by lupus-like autoimmune features and subsequent COVID-19 infection.

An older woman in her early 70s presented with fever, lethargy, weight loss, back pain, hypotension, raised inflammatory markers, cytopenia, markedly elevated lactate dehydrogenase (LDH), and progressive hypoxemia. She was initially treated for presumed sepsis, but repeated microbiological investigations and serial imaging did not identify an infectious source, and clinical improvement was not sustained. A transient malar rash and a positive antinuclear antibody raised concern for a lupus-like autoimmune disease; however, testing for anti-double-stranded DNA and extractable nuclear antigen was negative, and the broader autoimmune workup did not support systemic lupus erythematosus as the unifying diagnosis. Subsequent severe acute respiratory syndrome coronavirus 2 infection further complicated the interpretation of hypoxemia and deterioration. Despite antimicrobial therapy, corticosteroids, antiviral treatment, and supportive care, she developed progressive multiorgan failure and died approximately six weeks after admission.

Postmortem examination revealed widespread multiorgan IVLBCL involving the lungs, heart, kidneys, liver, spleen, and multiple additional extranodal sites. Pulmonary capillary involvement provided a clinicopathological explanation for persistent unexplained hypoxemia. This case demonstrates how IVLBCL may remain concealed when several plausible diagnoses coexist. Persistent culture-negative fever, constitutional decline, cytopenia, high LDH, nondiagnostic imaging, treatment nonresponse, and unexplained hypoxemia should prompt consideration of IVLBCL and early tissue-based investigation.

## Introduction

Intravascular large B-cell lymphoma (IVLBCL) is a rare and aggressive subtype of extranodal diffuse large B-cell lymphoma, characterized by the selective proliferation of malignant lymphoid cells within the lumina of small blood vessels, particularly capillaries and postcapillary venules [1,2]. Because the tumor remains predominantly intravascular, patients may lack lymphadenopathy, discrete tumor masses, circulating malignant cells, or specific early radiological findings [1-3].

The clinical presentation is heterogeneous and frequently overlaps with common medical conditions. Fever of unknown origin, constitutional symptoms, neurological or cutaneous features, cytopenias, elevated inflammatory markers, increased lactate dehydrogenase (LDH) levels, hypoxemia, and multiorgan dysfunction have all been described [3-6]. Consequently, IVLBCL may be mistaken for sepsis, vasculitis, autoimmune disease, occult infection, or nonspecific inflammatory disease, and the diagnosis is often delayed until advanced disease or autopsy [6-9]. Early recognition is important because rituximab-containing chemotherapy is associated with improved outcomes, particularly when the diagnosis is made before irreversible multiorgan failure [10,11]. Random deep skin biopsy, particularly when the tissue includes subcutaneous fat and bone marrow examination, may support the diagnosis even in the absence of visible skin lesions or lymphadenopathy [12-16].

We report an autopsy-proven case of disseminated IVLBCL in which culture-negative sepsis, lupus-like autoimmune features, and subsequent COVID-19 each provided plausible but incomplete explanations for the clinical course. The central diagnostic lesson is that IVLBCL is unlikely to be obvious at presentation; however, persistent atypical features, such as cytopenias, elevated LDH, constitutional decline, nondiagnostic investigations, treatment nonresponse, and unexplained hypoxemia, should prompt reconsideration of underlying hematological processes.

## Case presentation

An older woman in her early 70s presented with generalized malaise, intermittent fever, reduced appetite, progressive lethargy, and lower back pain. Her medical history included stage III chronic kidney disease, hypertension, diverticulosis, hiatus hernia, and a previous right hip replacement. On admission, her temperature was 38.1°C, blood pressure was 90/54 mmHg, heart rate was 116 bpm, and oxygen saturation was 93% on room air. She appeared pale and mildly confused. Collateral history revealed a gradual decline in cognitive function over several months and significant unintentional weight loss over the preceding four weeks. There was no rash, lymphadenopathy, joint swelling, organomegaly, or clinically apparent infectious focus.

Initial investigations demonstrated anemia, leukopenia, marked lymphopenia, elevated inflammatory markers, and markedly elevated LDH levels. Initial blood and urine cultures showed no growth. The key longitudinal laboratory abnormalities during admission are summarized in Table 1.

**Table 1:** High-yield longitudinal laboratory abnormalities during admission

Parameter	Reference range	Early/first available result	Peak, nadir, or later result	Diagnostic relevance
Hemoglobin	115-165 g/L	91 g/L	Nadir 73 g/L	Persistent anemia despite supportive treatment; part of the unresolved systemic/hematological signal
White cell count	4.0-11.0 × 10^9^/L	2.25 × 10^9^/L	Nadir 1.51 × 10^9^/L	Persistent leukopenia not explained by a single infective episode
Lymphocytes	1.0-4.5 × 10^9^/L	0.49 × 10^9^/L	Nadir 0.14 × 10^9^/L	Profound and persistent lymphopenia; a key discordant clue
Platelets	150-450 × 10^9^/L	178 × 10^9^/L	Fell to 80 × 10^9^/L late in admission	Evolving thrombocytopenia during progressive systemic deterioration
C-reactive protein	0-5 mg/L	81.8 mg/L	Peak 99.7 mg/L; 61.1 mg/L near terminal deterioration	Persistent inflammatory syndrome despite negative cultures and no sustained antimicrobial response
Procalcitonin	0.0-0.5 ug/L	0.44 ug/L	0.09-0.26 ug/L on later testing	Low/normal values made bacterial sepsis an incomplete unifying diagnosis
Lactate dehydrogenase	135-214 U/L	783 U/L	756 and 702 U/L on repeat testing	Persistently >700 U/L; important clue to high cell turnover or occult hematological malignancy
Ferritin	15-200 ug/L	418-466 ug/L	-	Inflammatory/malignancy signal; supportive but nonspecific
Albumin	35-50 g/L	28 g/L	Nadir 16 g/L	Progressive hypoalbuminemia reflecting severe systemic illness and inflammation
Lactate	Arterial 0.0-1.3 mmol/L; venous 0.0-2.3 mmol/L	Arterial lactate 3.3 mmol/L	Arterial lactate 6.3 mmol/L; venous lactate 4.6 mmol/L near terminal deterioration	Recurrent lactic acidosis during episodes of clinical deterioration

The combination of fever, hypotension, tachycardia, and elevated inflammatory markers led to initial treatment for presumed sepsis with intravenous fluids and broad-spectrum antimicrobial therapy. Constitutional symptoms, cytopenia, and markedly elevated LDH levels raised the possibility of an occult hematological process. This possibility was less apparent because there was no lymphadenopathy, organomegaly, circulating abnormal cells, or definite radiological evidence of malignancy.

Despite antimicrobial therapy, she continued to experience intermittent fever, progressive lethargy, and increasing oxygen requirements. Repeated blood and urine cultures, viral testing, cerebrospinal fluid analysis, and serial imaging failed to identify a convincing infectious source. Computed tomography pulmonary angiography excluded pulmonary embolism but demonstrated bilateral pulmonary nodules of uncertain significance (Figure 1A). Computed tomography revealed an indeterminate hepatic lesion but no definite evidence of malignancy (Figure 1B). Magnetic resonance imaging (MRI) of the brain showed bilateral hippocampal atrophy consistent with chronic neurodegenerative changes (Figures 1C, 1D), and MRI of the spine did not identify an alternative cause for her symptoms (Figure 1E). Transthoracic echocardiography revealed mild-to-moderate aortic stenosis with preserved systolic function and no evidence of infective endocarditis (Figure 2A). No cardiac shunt was identified on bubble echocardiography (Figure 2B). Lumbar puncture was unremarkable. Sepsis remained a plausible working diagnosis; however, microbiological investigations remained negative, and there was no sustained clinical response.

**Figure 1:**
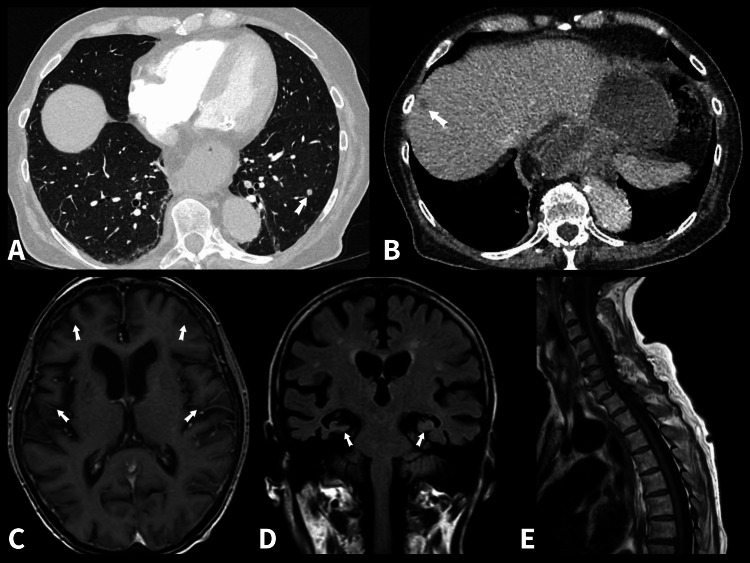
Multimodal imaging findings during investigation of refractory hypoxemia and fever of unknown origin (A) Axial CT pulmonary angiogram demonstrating a small left lower lobe pulmonary nodule (arrow), representative of multiple bilateral pulmonary nodules. (B) Contrast-enhanced CT abdomen showing a focal hypodense lesion within the liver (arrow). (C) Axial postcontrast T1-weighted MRI brain demonstrating frontotemporal atrophy (arrows). (D) Coronal FLAIR MRI brain showing bilateral hippocampal atrophy (arrows). (E) Sagittal T2-weighted MRI spine demonstrating no focal spinal cord lesion or compressive pathology CT: computed tomography; MRI: magnetic resonance imaging; FLAIR: fluid-attenuated inversion recovery

**Figure 2:**
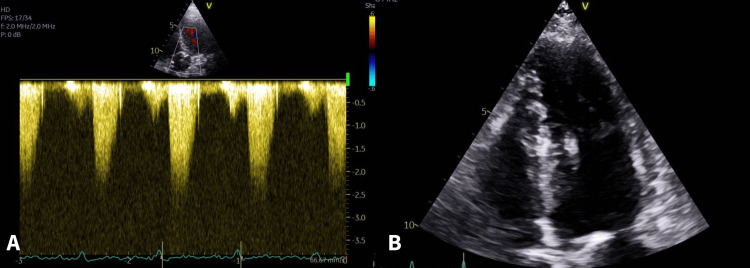
Echocardiographic findings (A) Continuous-wave Doppler interrogation across the aortic valve demonstrating elevated transvalvular velocities, in keeping with mild-to-moderate aortic stenosis. (B) Apical four-chamber transthoracic echocardiographic view during agitated saline contrast bubble study demonstrating opacification of the right-sided cardiac chambers without passage of microbubbles into the left-sided chambers, consistent with a negative bubble study and absence of a demonstrable right-to-left intracardiac shunt

During admission, she developed a faint malar rash, prompting an autoimmune evaluation. A positive antinuclear antibody (ANA) test shifted the diagnostic consideration toward systemic lupus erythematosus or another connective tissue disease, and oral prednisolone was initiated following a rheumatology review. However, testing for anti-double-stranded DNA antibody and extractable nuclear antigen was negative, and there was no convincing evidence of multisystem autoimmune disease. Corticosteroids were, therefore, reduced. Although the rash and positive ANA partially supported an autoimmune explanation, persistent fever, weight loss, cytopenia, elevated LDH, negative cultures, progressive lethargy, and ongoing hypoxia remained unexplained.

Hematological input was sought for persistent anemia and leukopenia. These were initially considered more consistent with reactive inflammatory changes than primary hematological disorders. Bone marrow examination was considered but not pursued at that stage because there was no lymphadenopathy, organomegaly, circulating malignant cells, or definitive radiological evidence of malignancy. Focused investigations relevant to the differential diagnosis are summarized in Table 2.

**Table 2:** Focused diagnostic investigations relevant to autoimmune, infectious, and hematological differentials ANA: antinuclear antibody; anti-B2GP: anti-beta-2 glycoprotein I antibody; CEA: carcinoembryonic antigen; CMV: cytomegalovirus; dsDNA: double-stranded DNA; EBV: Epstein-Barr virus; IVLBCL: intravascular large B-cell lymphoma; MPO: myeloperoxidase; PR3: proteinase 3; SLE: systemic lupus erythematosus; IgG: immunoglobulin G; IgM: immunoglobulin M; ANCA: antineutrophil cytoplasmic antibody; LDH: lactate dehydrogenase; ADENO: adenovirus; PCR: polymerase chain reaction

Diagnostic domain	Key available results	Interpretation
Autoimmune serology	ANA nuclear light intensity positive at 124 LIU, later 234 LIU; dsDNA negative on repeated testing: 2.4, 1.6, and 1.2 IU/mL	Supported lupus-like diagnostic consideration, but repeated negative dsDNA made definite SLE less convincing
Complement profile	C3 initially 1.02 g/L, transiently low at 0.62 g/L, later 0.98 and 0.93 g/L; C4 remained within range	Transient complement abnormality was nonspecific and did not establish active lupus as the unifying diagnosis
Vasculitis/antiphospholipid testing	MPO <1.0 IU/mL; PR3 <1.0 IU/mL; anticardiolipin IgG 1.6, IgM 2.6; anti-B2GP IgG 1.4, IgM <2.9; lupus anticoagulant ratio 1.10	Did not support ANCA-associated vasculitis or antiphospholipid syndrome
Anemia work-up	Folate 2.8 ug/L; iron 2.4-3.1 umol/L; transferrin saturation 7%-10%; ferritin 418-466 ug/L; B12 746 ng/L	Demonstrated deficiency/inflammatory pattern, but did not explain the full syndrome of fever, cytopenia, high LDH, and hypoxia
Direct antiglobulin/hemolysis markers	Direct Coombs/anti-IgG positive 2+, later 3+; reticulocyte count 118 × 10⁹/L; haptoglobin 2.85 g/L	Suggested immune red-cell reactivity, but raised haptoglobin argued against this being a complete hemolytic explanation
Immunoglobulins/light chains	IgA 7.32 then 6.14 g/L; IgM 2.66 then 2.30 g/L; IgG 16.3 then 13.6 g/L; free kappa and lambda elevated but kappa ratio normal at 1.25 and 0.94	Favored polyclonal inflammatory activation rather than plasma-cell dyscrasia
Infection-related biomarkers	Procalcitonin remained low/normal; ADENO/CMV/EBV PCR viral load <1,000 IU/mL	Did not support bacterial sepsis or systemic viral infection as the sole explanation
Tumor markers	CEA 1.8 ug/L; CA125 13 kU/L; CA15-3 16 KU/L; CA19-9 7 kU/L	Noncontributory; normal tumor markers did not exclude lymphoma, particularly IVLBCL

Approximately two weeks after admission, she tested positive for COVID-19 using polymerase chain reaction (PCR) and was treated with dexamethasone and remdesivir. Procalcitonin levels remained within the normal range. Computed tomography pulmonary angiography excluded pulmonary embolism but demonstrated bilateral pulmonary nodules of uncertain significance. A bubble echocardiogram excluded an intracardiac shunt, and tumor markers, including carcinoembryonic antigen, cancer antigen (CA)-125, CA 19-9, and alpha-fetoprotein, were within normal limits. COVID-19 provided a competing explanation for hypoxemia and systemic inflammation; however, it did not explain the preceding weight loss, cytopenias, elevated LDH, culture-negative fever, or lack of sustained clinical improvement. In retrospect, the persistence of hypoxia despite exclusion of pulmonary embolism, significant cardiac dysfunction, and intracardiac shunt was consistent with an occult pulmonary microvascular process.

Over the following weeks, the patient developed worsening hypoxemia, recurrent hypotension, rising inflammatory markers, acute kidney injury, fluctuating consciousness, and progressive functional decline despite antimicrobial, corticosteroid, and antiviral therapy, as well as supportive care. A positron emission tomography scan was planned but could not be performed due to clinical deterioration. Recurrent episodes of hypotension and hypoxia were treated with further broad-spectrum antibiotics and intravenous fluids; however, her condition continued to worsen. A do-not-attempt-resuscitation decision was made, and she died approximately six weeks after admission, following profound hypotension and respiratory failure. The patient’s clinical course, evolving diagnostic considerations, and persistent unresolved clues are summarized in Figure 3.

**Figure 3:**
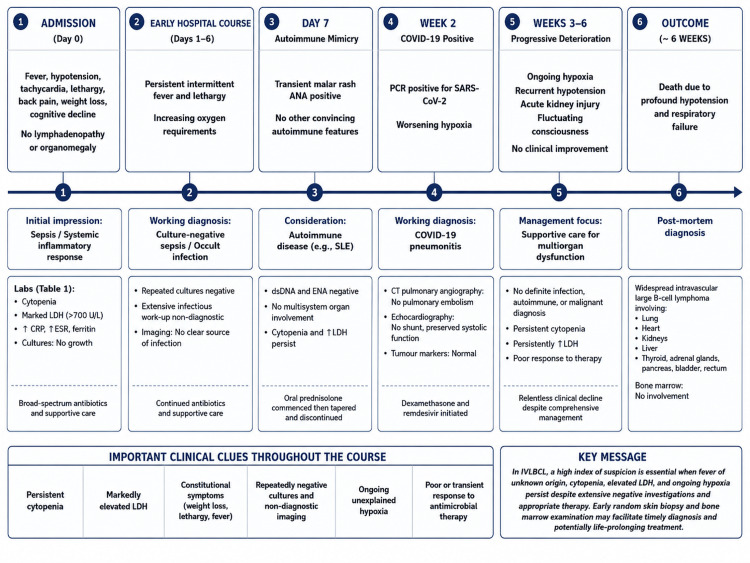
Summary timeline of the patient’s clinical course, highlighting important diagnostic clues and the key learning message from the case Clinical timeline summarizing the patient’s progression from admission with culture-negative sepsis-like illness, lupus-like autoimmune features, and subsequent SARS-CoV-2 positivity to progressive deterioration and postmortem diagnosis of disseminated intravascular large B-cell lymphoma ANA: antinuclear antibody; CRP: C-reactive protein; dsDNA: anti-double-stranded DNA; ENA: extractable nuclear antigen; ESR: erythrocyte sedimentation rate; IVLBCL: intravascular large B-cell lymphoma; LDH: lactate dehydrogenase; PCR: polymerase chain reaction; SARS-CoV-2: severe acute respiratory syndrome coronavirus 2; SLE: systemic lupus erythematosus; CT: computed tomography Image credit: The image was created by the authors Khalid Shahzad and Muhammad Tanseer Sibtain Raza using Canva (Canva Pty Ltd, Sydney, Australia)

Postmortem examination revealed no macroscopic tumor mass, deep lymphadenopathy, or occlusive coronary thrombosis. The lungs were markedly congested and edematous, with small bilateral pleural effusions; however, there was no histological evidence of pneumonia. The heart showed coronary atherosclerosis, patchy ischemic myocardial fibrosis, and myocyte hypertrophy. The liver showed moderate fatty change without a focal lesion. The kidneys were mildly atrophic with hypertensive vascular changes and mild age-related glomerulosclerosis; however, there was no crescentic nephritis. The spleen was of normal size and appearance, and the brain showed no gross abnormalities.

Histological examination revealed widespread IVLBCL involving the lungs, heart, kidneys, liver, thyroid, adrenal glands, pancreas, spleen, bladder, and rectum. Neoplastic lymphoid cells were predominantly confined to small blood vessels and sinusoids. Immunohistochemistry demonstrated a B-cell phenotype, with tumor cells positive for CD20 and CD79a and negative for CD3. CD34 staining confirmed intravascular localization, and weak variable MUM-1 staining was present. In the lungs, numerous atypical lymphoid cells were identified within alveolar septal capillaries, providing a clinicopathological explanation for persistent, otherwise unexplained hypoxemia (Figure 4). In the heart, atypical lymphoid cells were present within blood vessels, raising the possibility of a cardiac contribution to terminal deterioration. Bone marrow showed preserved trilineage hematopoiesis without lymphomatous infiltration, emphasizing that marrow involvement is not required for disseminated IVLBCL. The postmortem organ distribution is summarized in Table 3.

**Figure 4:**
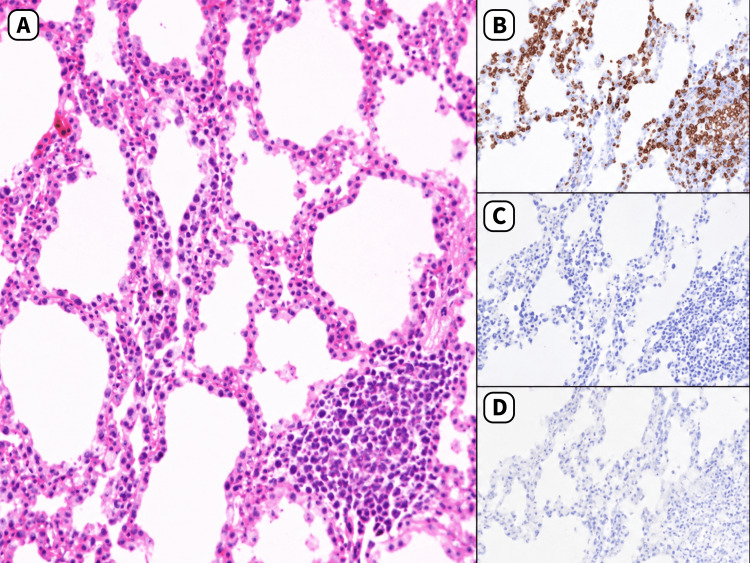
Pulmonary histopathology and immunohistochemistry supporting intravascular large B-cell lymphoma (A) Hematoxylin and eosin staining of lung tissue shows numerous atypical lymphoid cells predominantly within alveolar septal capillaries, with a small focus of extravascular infiltration. (B) CD20 immunohistochemistry highlights the atypical cells, confirming B-cell lineage. (C) CD3 staining is negative in the atypical cell population. (D) AE1/AE3 epithelial marker staining is negative, arguing against epithelial malignancy. These findings support pulmonary intravascular large B-cell lymphoma and provide a clinicopathologic explanation for the patient’s persistent unexplained hypoxia

**Table 3:** Postmortem organ involvement and clinicopathologic relevance IVLBCL: intravascular large B-cell lymphoma

Organ/system	Postmortem finding	Clinicopathological relevance
Lungs	Intravascular lymphoma within pulmonary capillaries; congestion and edema; no histological pneumonia	Explains persistent hypoxia despite nondiagnostic antemortem investigations
Heart	Intravascular lymphoma within cardiac vessels; coronary atherosclerosis; patchy ischemic myocardial fibrosis	Possible contribution to hypotension and terminal cardiovascular decline
Kidneys	Intravascular lymphoma; hypertensive vascular changes; mild age-related glomerulosclerosis; no crescentic nephritis	Supports lymphoma-related multiorgan disease; argues against lupus nephritis
Liver	Sinusoidal and small-vessel involvement; moderate fatty change; no focal lesion	Explains systemic disease despite absence of a liver mass
Spleen	Atypical cells within small sinusoidal vessels; normal size macroscopically	Demonstrates occult involvement despite no splenomegaly
Thyroid	Intravascular lymphoma in small vessels; atrophic thyroid	Additional occult extranodal involvement
Adrenal glands	Atypical lymphoid cells within blood vessels	Disseminated vascular disease
Pancreas	Atypical lymphoid cells within blood vessels	Multiorgan intravascular spread
Bladder	Numerous atypical lymphoid cells within submucosal vessels	Occult extranodal involvement
Rectum	Atypical lymphoid cells within submucosal vessels	Occult gastrointestinal involvement
Bone marrow	Preserved trilineage hematopoiesis; no lymphomatous infiltration	Important diagnostic caveat: marrow may be negative in disseminated IVLBCL

Respiratory viral testing confirmed COVID-19 positivity, but postmortem interpretation considered ongoing COVID-19 infection unlikely to have materially contributed to death. The final cause of death was recorded as multiorgan failure secondary to IVLBCL. Overall, the clinical course illustrates how several plausible diagnostic explanations, sepsis, lupus-like autoimmunity, and COVID-19, coexisted with persistent, unresolved clues to an occult intravascular lymphoma.

## Discussion

IVLBCL is diagnostically challenging because malignant B cells proliferate predominantly within small vascular lumina rather than forming nodal enlargement, tumor masses, or obvious circulating malignant cells [1-3]. Therefore, it has been described as an “oncologist’s great imitator” [4]. The present case illustrates a more specific clinical problem: diagnostic eclipse. Culture-negative sepsis, lupus-like autoimmunity, and COVID-19 each offered plausible explanations for part of the presentation, while persistent discordant clues continued to suggest an occult hematological process. This framing is clinically important because it shifts attention from rarity alone to the practical question of when persistent unexplained features should trigger tissue diagnosis.

The initial sepsis-focused approach was clinically appropriate for this patient. Fever, hypotension, tachycardia, frailty, and elevated inflammatory markers justified urgent antimicrobial therapy and supportive care. However, the subsequent clinical trajectory was atypical for an uncomplicated infection. Repeated cultures were negative, no convincing infectious focus was identified, and improvement with broad-spectrum antibiotics was transient. Simultaneously, constitutional decline, cytopenia, profound lymphopenia, and markedly elevated LDH suggested a process beyond infection. Large series and reviews of IVLBCL consistently describe fever, B symptoms, cytopenia, elevated LDH, and nonspecific inflammatory syndromes as common but easily overlooked clues [2,5-7]. Therefore, persistent culture-negative fever with cytopenia and LDH elevation should prompt reassessment for occult hematological disease when the infectious workup remains unrevealing.

Autoimmune mimicry formed the second diagnostic layer. The development of a transient malar rash and positive ANA understandably shifted attention toward systemic lupus erythematosus or another connective tissue disease. However, the autoimmune explanation remained incomplete: anti-double-stranded DNA and extractable nuclear antigen testing were negative, there was no convincing multisystem autoimmune pattern, and postmortem renal examination did not show features of active lupus nephritis. ANA positivity and autoimmune-like diagnostic pitfalls have been described in IVLBCL, although the evidence is limited to case-level reports [8]. Therefore, positive autoimmune serology should be interpreted in the context of the entire syndrome, particularly when cytopenia, elevated LDH, treatment nonresponse, and systemic decline remain unexplained.

Persistent hypoxemia was a major clinicopathological clue. In older medical inpatients, hypoxemia is commonly attributed to infection, pulmonary embolism, cardiac dysfunction, atelectasis, or viral pneumonitis. In this case, hypoxemia persisted despite nondiagnostic investigations and exclusion of several common mechanisms, including pulmonary embolism, significant systolic dysfunction, and intracardiac shunt. Pulmonary IVLBCL can cause respiratory failure through microvascular tumor occlusion without producing a discrete mass or characteristic radiological pattern. Similar cases have been diagnosed using transbronchial lung biopsy or random skin biopsy in patients with hypoxemia and minimal or normal chest imaging findings [9,16]. The postmortem finding of atypical lymphoid cells within the pulmonary capillaries explains why the oxygen requirement remained disproportionate to conventional antemortem investigations.

COVID-19 created the third diagnostic frame. Once the severe acute respiratory syndrome coronavirus 2 (SARS-CoV-2) PCR test became positive, it offered a plausible explanation for hypoxemia, inflammation, lymphopenia, and clinical deterioration. However, several features argued against COVID-19 as the unifying diagnosis: fever, weight loss, cytopenia, high LDH, and functional decline preceded SARS-CoV-2 detection; procalcitonin levels remained low or normal; pulmonary embolism and intracardiac shunt were excluded; and there was no sustained improvement with antimicrobial, corticosteroid, antiviral, and supportive treatment. A recent case report described IVLBCL as an option in the differential diagnosis after SARS-CoV-2 reinfection in the context of a presumed inflammatory neurological syndrome; however, the evidence remains limited, and any causal relationship is unclear [17]. In this patient, COVID-19 is best regarded as a diagnostic confounder rather than the primary cause of the overall clinical syndrome, as postmortem examination demonstrated no histopathological evidence of COVID-19 pneumonitis.

The postmortem findings explain why conventional investigations were unrevealing. There was no macroscopic tumor mass, significant lymphadenopathy, or clear radiological target. Instead, lymphoma cells were distributed within small vessels and sinusoids across multiple organs, including the lungs, heart, kidneys, liver, spleen, thyroid, adrenal glands, pancreas, bladder, and rectum. This pattern is characteristic of IVLBCL and accounts for its ability to cause severe systemic illness without classical imaging evidence of malignancy [1-3]. The absence of bone marrow involvement is also instructive; a negative marrow assessment does not exclude IVLBCL, and complementary tissue strategies are required when clinical suspicion persists.

The key clinical question is when to pursue a tissue diagnosis. Random skin biopsy has emerged as a valuable diagnostic tool for suspected IVLBCL, even in patients without visible skin lesions [12,13]. The diagnostic yield improves with sampling multiple sites and obtaining tissue deep enough to include the subcutaneous fat [13-15]. In this case, the convergence of persistent culture-negative fever, constitutional decline, cytopenia, LDH repeatedly above 700 U/L, nondiagnostic imaging, treatment nonresponse, and unexplained hypoxemia now represents a reasonable threshold for random deep skin biopsy, with bone marrow examination as a complementary investigation. This recommendation is retrospective and should not imply that IVLBCL was immediately apparent at presentation; rather, it highlights the point at which repeated discordant clues should prompt a lower threshold for tissue diagnosis.

This case illustrates systemic diagnostic complexity rather than individual error. Anchoring to sepsis was reasonable at presentation, considering that an autoimmune disease was reasonable after the development of a rash and ANA positivity, and attributing part of the respiratory deterioration to COVID-19 was understandable. The challenge was the cumulative effect of sequential plausible explanations, each of which reduced the visibility of discordant clues. The cognitive diagnostic error literature emphasizes the need for reassessment when the clinical course does not follow the expected trajectory [18]. In this patient, the repeated discordant clues were culture-negative fever, cytopenia, high LDH, constitutional decline, nondiagnostic imaging, treatment nonresponse, and persistent hypoxemia.

Early recognition is clinically important because IVLBCL is aggressive but potentially treatable. Rituximab-containing chemotherapy has improved outcomes compared with historical experience, particularly when the diagnosis is made before irreversible multiorgan failure [10,11]. Unfortunately, delayed diagnosis remains common because IVLBCL often lacks lymphadenopathy, mass lesions, circulating malignant cells, or an obvious biopsy target. In older patients with persistent culture-negative inflammatory illness, unexplained cytopenia, elevated LDH, nondiagnostic imaging, and hypoxemia without a clear cause, IVLBCL should remain in the differential diagnosis, even when sepsis, autoimmune disease, or COVID-19 appear to offer partial explanations.

This case report has some limitations. The diagnosis was established postmortem; therefore, the proposed antemortem diagnostic triggers were retrospective. Random skin biopsy, bone marrow examination, and positron emission tomography were not performed before death; therefore, their diagnostic yield in this patient could not be determined. Histological images were limited to representative pulmonary sections, whereas the full extent of multiorgan involvement was summarized from the official postmortem report. COVID-19 may have contributed to hypoxemia and systemic decline, although the broader syndrome preceded SARS-CoV-2 positivity, and postmortem findings supported IVLBCL as the primary cause of death. This report aims to highlight a reproducible pattern of persistent clues that may justify an earlier tissue diagnosis in similar cases.

## Conclusions

This case demonstrates a diagnostic eclipse in fatal IVLBCL, in which culture-negative sepsis, lupus-like autoimmune features, and COVID-19 each provided plausible but incomplete explanations for the clinical course. Persistent cytopenia, markedly elevated LDH, constitutional decline, negative microbiological investigations, nondiagnostic imaging, poor treatment response, and unexplained hypoxia remained key clues to occult intravascular lymphoma. In similar cases, early random deep skin biopsy, including subcutaneous fat, with bone marrow examination as a complementary investigation, where clinically appropriate, should be considered before irreversible multiorgan deterioration occurs.
